# Adaptive Responses Limited by Intrinsic Noise

**DOI:** 10.1371/journal.pone.0136095

**Published:** 2015-08-25

**Authors:** Prabhat Shankar, Masatoshi Nishikawa, Tatsuo Shibata

**Affiliations:** 1 Department of Mathematical and Life Sciences, Hiroshima University, Higashi-Hiroshima, Japan; 2 Laboratory for Physical Biology, RIKEN Quantitative Biology Center, Kobe, Japan; 3 RIKEN Center for Developmental Biology, Kobe, Japan; Centrum Wiskunde & Informatica (CWI) & Netherlands Institute for Systems Biology, NETHERLANDS

## Abstract

Sensory systems have mechanisms to respond to the external environment and adapt to them. Such adaptive responses are effective for a wide dynamic range of sensing and perception of temporal change in stimulus. However, noise generated by the adaptation system itself as well as extrinsic noise in sensory inputs may impose a limit on the ability of adaptation systems. The relation between response and noise is well understood for equilibrium systems in the form of fluctuation response relation. However, the relation for nonequilibrium systems, including adaptive systems, are poorly understood. Here, we systematically explore such a relation between response and fluctuation in adaptation systems. We study the two network motifs, incoherent feedforward loops (iFFL) and negative feedback loops (nFBL), that can achieve perfect adaptation. We find that the response magnitude in adaption systems is limited by its intrinsic noise, implying that higher response would have higher noise component as well. Comparing the relation of response and noise in iFFL and nFBL, we show that whereas iFFL exhibits adaptation over a wider parameter range, nFBL offers higher response to noise ratio than iFFL. We also identify the condition that yields the upper limit of response for both network motifs. These results may explain the reason of why nFBL seems to be more abundant in nature for the implementation of adaption systems.

## Introduction

Sensory systems have a number of mechanisms to keep track of their environment and to respond appropriately to any changes. Adaptation is one such mechanism where the system shows a transient response to a temporal perturbation followed by a return back to its original state [1–5]. Such adaptive response contributes to detecting stimulus over a wide dynamic range of environmental conditions [6]. By responding to perturbations, adaptation systems can also perform sensing of temporal changes in stimulus. Such a temporal sensing capability of adaptation system is used in bacterial chemotaxis [7–11]. An adaptive response has also been shown to occur in many eukaryotic systems, such as yeast osmoregulation [12], olfactory receptor cells [13] and calcium homeostasis [14]. In chemotactic cell *Dictyostelium*, an adaptive response is used to sense the direction of wave propagation in the chemoattractant concentration field [15–17]. While these diverse systems show adaptive response, common principles underlie their mechanism. It has been reported that the perfect adaptation can be achieved by two types of network motifs [18]. One is incoherent feedforward loop (iFFL), and the other is negative feedback loop (nFBL). For instance, the chemotaxis receptor in bacteria uses feedback mechanism to achieve perfect adaptation [19], whereas the adaptive response in chemotaxis signaling pathway of *Dictyostelium* cells is operated by a feed-forward mechanism [20].

Adaptive responses are known to be extremely sensitive to small changes in the stimulus [21]. Such a sensitive response can be achieved by amplification mechanisms, such as molecular cooperativities among subunits and a push-pull type reaction of antagonistic enzymes [22, 23]. Perfect adaptation systems have a larger tendency towards more sensitive response as compared with imperfect adaptation [24]. Generally, sensitive amplification mechanisms are also sensitive to stochastic variation in the stimulus, and they may further generate large stochastic variations in their activities [25]. Because the cellular reactions are often operated by a small number of molecules, such stochastic fluctuations are inevitable. For a relatively simple reaction that works under the thermal equilibrium conditions and reactions which work essentially with one degree of freedom, a relation between fluctuations and responses has been known. For non-equilibrium condition, relations between fluctuation and responses at steady state values has been also studied (see [26] and references cited therein). Adaptive mechanism works far from thermodynamic equilibrium and thus adaptive response requires free energy input [27, 28]. Therefore, a simple relation between response and fluctuation may not be expected as in the situations close to thermodynamic equilibrium.

**Fig 1:**
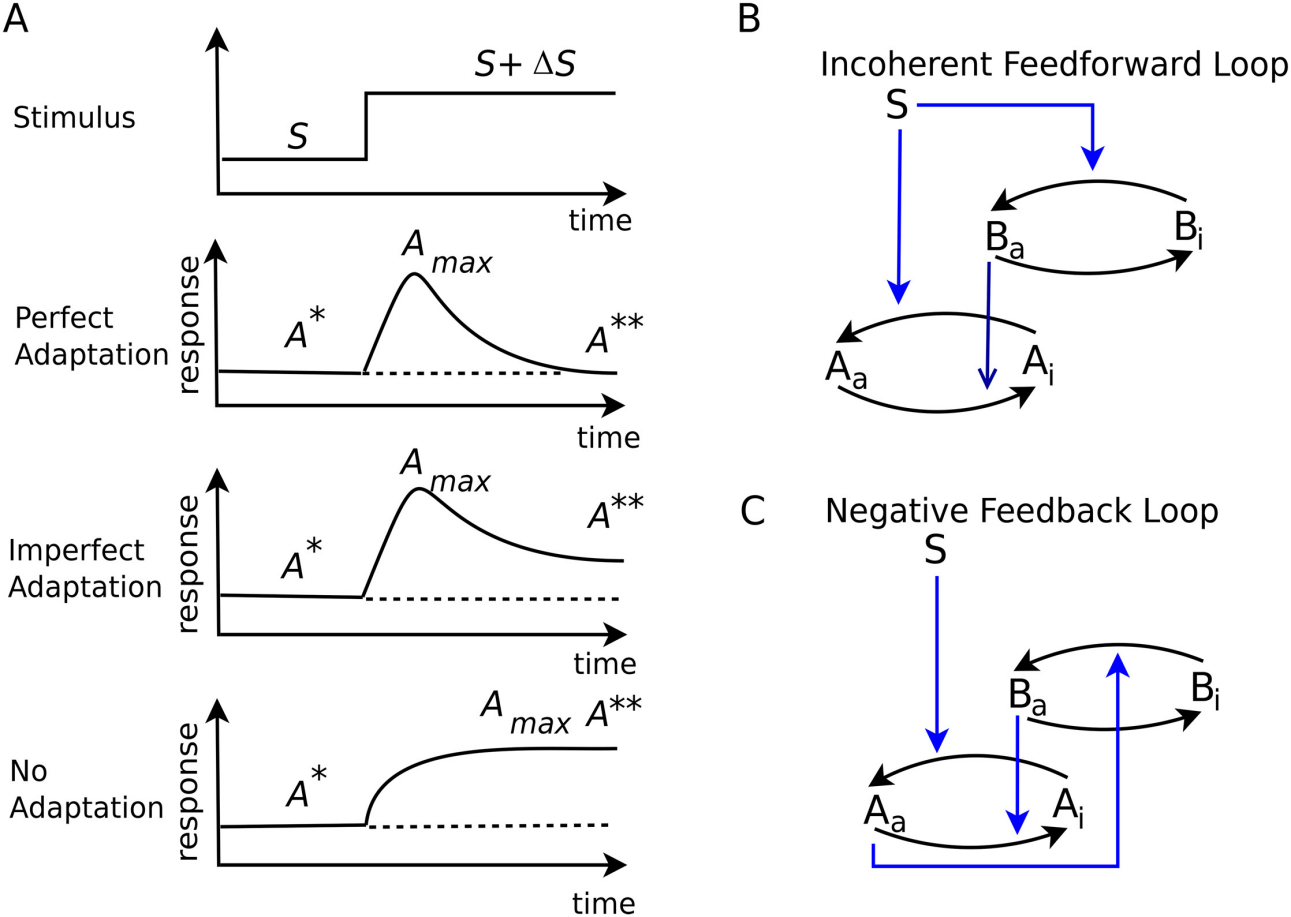
Adaptive responses and network motifs capable of perfect adaptation. (A) For an input perturbation to a system in steady state, three types of response is possible: perfect adaptation, where the system comes back exactly to the pre stimulus state, imperfect adaptation where it returns partially towards the prestimulus state, and no adaptation, where it remains in its new state. The two network motifs can show perfect adaptation to perturbations: (B) Incoherent feedforward loop(iFFL) and (C) negative feedback loop(nFBL). Both species A and B have an active (A_a_) and inactive state(A_i_), which can inter-convert between each other through enzymatic reactions. The number of A_a_ is considered to be the output.

Previously, a linear relationship between behavioral variability and response time in bacterial chemotaxis has been found experimentally [29]. A theoretical study indicates that such a linear relationship can hold in a limited parameter range, otherwise a combination of fluctuations and nonlinearity generally produces a complex response with many time scales, which deviates from the linear relationship [30]. A relation between noise suppression and responsiveness in feedback loops for non adaptive systems has been reported previously [31, 32]. For adaptive networks, their noise filtering [33, 34], noise generation and noise propagation characteristic [35, 36] have been studied. However, how the relation between response in adaptation system, and its steady state fluctuations before perturbations has not been explored yet. Such a relation can be important to understand the limitations of adaptive response in a noisy enviornment. While with a high response magnitude, smaller perturbation can be detected by the adaptive system, it becomes difficult to distinguish between small random fluctuations and deterministic changes in the presence of noise. Therefore, we study the quantitative relation between the peak response and fluctuations before perturbation in adaptation reactions. Here, we focus on the intrinsic noise of the adaptive response, that is the noise generated in the adaptation reaction, and ignore the fluctuations in the input perturbation. Thus, in this paper, we consider the adaptive network motifs of iFFL and nFBL that consist of two signaling molecules as shown in Fig 1(B) and 1(C). Using numerical simulation and theoretical analysis, we first show that the peak amplitude in the adaptive response is limited by noise. We then study the conditions when the peak amplitude achieves its upper limit, and also study the key differences between iFFL and nFBL in this context.

## Results

### Adaptation network models of two enzymes

We study iFFL and the nFBL consisting of three nodes, where the input node is S, the output node is A and the intermediate node is B. For the nodes A and B, we consider enzymes which can take active and inactive states. We denote the active form by A_a_ and B_a_ and the inactive form by A_i_ and B_i_, respectively. These states can inter convert into each other by enzymatic reactions. Thus, the total numbers of molecules of A and B, denoted by *N*_A_ and *N*_B_, respectively, are constant in time. For the sake of convenience, the numbers of A_*a*_, B_*a*_ and S are denoted by *A*, *B* and *S*, respectively.

In iFFL, the input node S activates both A and B, and the active form B_a_ inactivates A_a_ (incoherent feedforward loop, Fig 1B). Thus, S regulates A through two regulatory pathway with opposing regulatory effects. The inactivation of B is carried out by a basal enzyme whose number remains fixed, and whose concentration we assume to be unity. This reaction scheme can be represented by

$${\text{A}}_{\text{i}}\overset{\Gamma_{a}^{\text{A}}\times S}{\underset{\Gamma_{d}^{\text{A}}\times B}{⇌}}{\text{A}}_{\text{a}},\;{\text{B}}_{\text{i}}\overset{\Gamma_{a}^{\text{B}}\times S}{\underset{\Gamma_{d}^{\text{B}}}{⇌}}{\text{B}}_{\text{a}}$$ (1)

where the rates of reaction per molecule for activation and deactivation, for the species A and B, are given by $\Gamma_{a}^{\text{A}}\times S$, $\Gamma_{d}^{\text{A}}\times B$, $\Gamma_{a}^{\text{B}}\times S$ and $\Gamma_{d}^{\text{B}}$, respectively.

In nFBL, the input node S regulates only the output node A (Fig 1B). While A activates B, node B inactivates A, completing the negative feedback loop. Node B decays to the inactive form by the basal enzyme whose number is fixed with unity concentration. The reaction scheme for the nFBL can thus be written as:

$${\text{A}}_{\text{i}}\overset{\Gamma_{a}^{\text{A}}\times S}{\underset{\Gamma_{d}^{\text{A}}\times B}{⇌}}{\text{A}}_{\text{a}},\;{\text{B}}_{\text{i}}\overset{\Gamma_{a}^{\text{B}}\times A}{\underset{\Gamma_{d}^{\text{B}}}{⇌}}{\text{B}}_{\text{a}}$$ (2)

These reaction rates are described by the Michaelis-Menten kinetics. For instance, the total activation rate for species A for the iFFL can be written as

$$\begin{array}{c} \Gamma_{a}^{A}S\left(N_{A}-A\right)=V_{\text{SA}}S\frac{N_{A}-A}{K_{\text{SA}}+\left(N_{A}-A\right)} \end{array}$$ (3)

where *V*_SA_ is the maximum reaction rate, and *K*_SA_ is the Michaelis constant giving the half maximum concentration. The reaction rate for the deactivation can be written similarly. The total reaction rates are given by

$$\Gamma_{a}^{A}S\left(N_{A}-A\right)=\frac{V_{\text{SA}}S\left(N_{A}-A\right)}{K_{\text{SA}}+N_{A}-A},$$ (4)

$$\Gamma_{d}^{A}B\cdot A=\frac{V_{\text{BA}}B\cdot A}{K_{\text{BA}}+A},$$ (5)

$$\Gamma_{d}^{B}B=\frac{V_{B}B}{K_{B}+B}.$$ (6)

For the activation of B, the rate for iFFL is given by

$$\Gamma_{a}^{B}S\left(N_{B}-B\right)=\frac{V_{\text{SB}}S\left(N_{B}-B\right)}{K_{\text{SB}}+N_{B}-B},$$ (7)

whereas for nFBL

$$\Gamma_{a}^{B}A\left(N_{B}-B\right)=\frac{V_{\text{AB}}A\left(N_{B}-B\right)}{K_{\text{AB}}+N_{B}-B}.$$ (8)

We shall denote the steady state numbers of A and B by A* and B* respectively.

For iFFL, perfect adaptation is achieved if the number of intermediate node B is proportional to the level of input S. If we choose the parameters such that *N*_*B*_ − *B* ≪ *K*_SB_, and *K*_B_ ≪ *B*, then, with the constants $\Gamma_{\text{a}}^{\text{B}}\approx V_{\text{SB}}/K_{\text{SB}}$ and $\Gamma_{\text{d}}^{\text{B}}\approx V_{\text{B}}/K_{\text{B}}$, B becomes proportional to the input S.

$$B^{*}≅\frac{S\Gamma_{a}^{B}}{\Gamma_{d}^{B}}$$ (9)

This makes the number of node A independent of the input, as it is activated by one pathway and simultaneously deactivated by the other.

In the nFBL given by Eqs (2), (4), (5), (6), and (8), the perfect adaptation of A_a_ is achieved when the reaction rates for B, Eqs (6) and (8), do not depend on the number of B at the reaction steady state. Such a situation is obtained if *K*_*B*_ ≪ *B* and *K*_*AB*_ ≪ *N*_*B*_ − *B* in Eqs (6) and (8), respectively. Then, the reaction equilibrium in B gives *V*_*B*_ = *AV*_*AB*_, leading towards A independent of S, as

$$A^{*}≅\frac{V_{B}}{V_{AB}}\;\text{and}\;\Gamma_{a}^{A}S\left(N_{A}-A^{*}\right)=\Gamma_{d}^{A}A^{*}B^{*}$$ (10)

### The correlation between gain and noise in adaptation reactions

**Fig 2:**
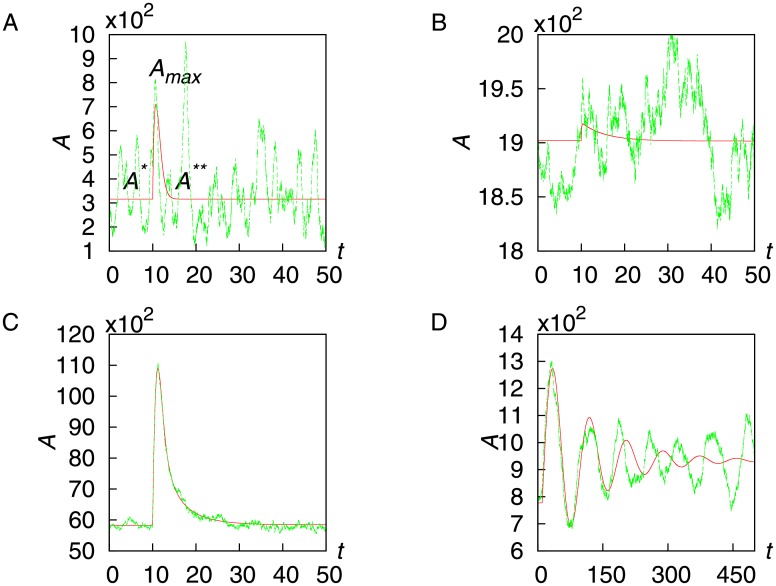
Stochastic reactions with perfect adaptation response. For a randomly selected network in steady state, a unit step input stimulus was applied at *t* = 10. Fig 2(A-B) shows the response for iFFL, while Fig 2(C-D) shows the non-oscillatory and damped oscillatory response for nFBL respectively. The green lines show the response of the network obtained using Gillespie Algorithm, while red lines show the corresponding deterministic response obtained from differential equations. The parameters for each case are provided in the section *Methods*.

When a step stimulus is applied, the adaptive network shows a response by changing its activity *A*, which is followed by adaptation through returning towards the prestimulus level. The magnitude of this response varies for different parameters. In Fig 2, the time series of four different parameter sets are plotted for iFFLs (A-B) and nFBL (C-D). For each of these networks in steady state, a unit step perturbation of magnitude Δ*S* = *S* was provided at time *t* = 10. The green lines show the time series obtained by stochastic simulation using Gillespie Algorithm [37]. For a guide, the time series obtained by the corresponding differential equation are plotted as well(red line). Among the two examples from iFFLs in Fig 2(A) and 2(B), the response in Fig 2(A) is slightly distinguishable, while in Fig 2(B), it is completely blurred by noise. In contrast, the response in Fig 2(C) is clearly distinguishable over the noise. Depending on the parameter value, nFBL can show both non-oscillatory as well as oscillatory response, as shown in Fig 2(C) and 2(D) respectively. iFFL shows only non-oscillatory responses.

Using the time series of the responses above, the response magnitude can be quantified by gain *g*, which is defined as a ratio between the fractional changes in the input signal *S* and in output signal *A*, that is,

$$g=\frac{\left|\Delta A\right|/A^{*}}{\left|\Delta S\right|/S}.$$ (11)

where Δ*A* is the change in *A* from the steady state value A* before external stimulus to the maximum response *A*_max_ (see Fig 2A), when the signal intensity is changed from *S* to *S* + Δ*S*. For this quantification, we used A* and *A*_max_ obtained by the differential equation without stochastic effects. Noise can be quantified by the variance $\sigma_{a}^{2}$ of time series of *A* at the steady state using the Gillespie algorithm. The variance is usually proportional to the average, we consider the ratio between the variance and the average, which is called Fano factor *f* defined as

$$f=\sigma_{a}^{2}/A^{*}.$$ (12)

We note here that the average and the variance are measured for the number of molecules instead of concentrations.

The exhibited response for a given parameter can be classified into three groups using (A*, *A*_max_, A**), where A** is the steady state after the stimulus as shown in Fig 1A. The first group shows perfect adaptation with ∣*A*_max_ − A*∣ > 0 and A* ≈ A**. The second group shows adaptive response which is not perfect, that is ∣*A*_max_ − A*∣ > 0 and A** ≠ A*. The last group does not show adaptive response with *A*_max_ ≈ A**. The tendency of network to return to its prestimulus state after a response is quantified by the adaptation tendency *α*, given by

$$\alpha =\frac{A_{\text{max}}-A^{**}}{A_{\text{max}}-A^{*}}$$ (13)

Here, we classify the response into the first two groups with adaptive tendency when *α* ≥ 0.2, whereas the response is non adaptive when *α* < 0.2. The precision of perfect adaptation is quantified by the adaptation error *ϵ*, given by

$$\epsilon =\frac{|A^{**}-A^{*}|/A^{*}}{|\Delta S|/S}$$ (14)

Here, we consider that the system shows the perfect adaptation when *ϵ* < 0.01.

To study the relation between response and noise, we quantified the gain and Fano factor over 10^6^ parameters for both iFFL and nFBL. Here, we focus on the parameters that show only adaptive responses (the first and second groups), and exclude the non adaptive parameters for which (*α* < 0.2). These networks have eight different kinetic parameters, as introduced in the last section. Each of them were sampled from a uniform distribution on a logarithmic scale from the range [10^−3^,10^3^] using random sampling(see Methods for detail). We also excluded those parameters from our analysis for which either the steady state was too small or too large, that is {(*A*/*V*, *B*/*V*, (*N*_*A*_ − *A*)/*V*, (*N*_*B*_ − *B*)/*V*) <.001}, where *V* = 10,000 is the system volume. We also removed those parameters for which the gain was too small, *i.e.* *g* <.001. For iFFL, 8.3% parameters among 10^6^ randomly chosen parameters showed adaptive response, among which 41% showed perfect adaptation and 59% were imperfect. For nFBL, 12.8% showed adaptive response, among which 5.3% showed perfect adaptation while 94.7% were imperfect. Among the parameters with adaptive responses, 56.64% showed non oscillatory response, and 43.35% showed damped oscillation. Among the parameters with perfect adaptation, 43.56% was non-oscillatory while 56.44% showed damped oscillation.

**Fig 3:**
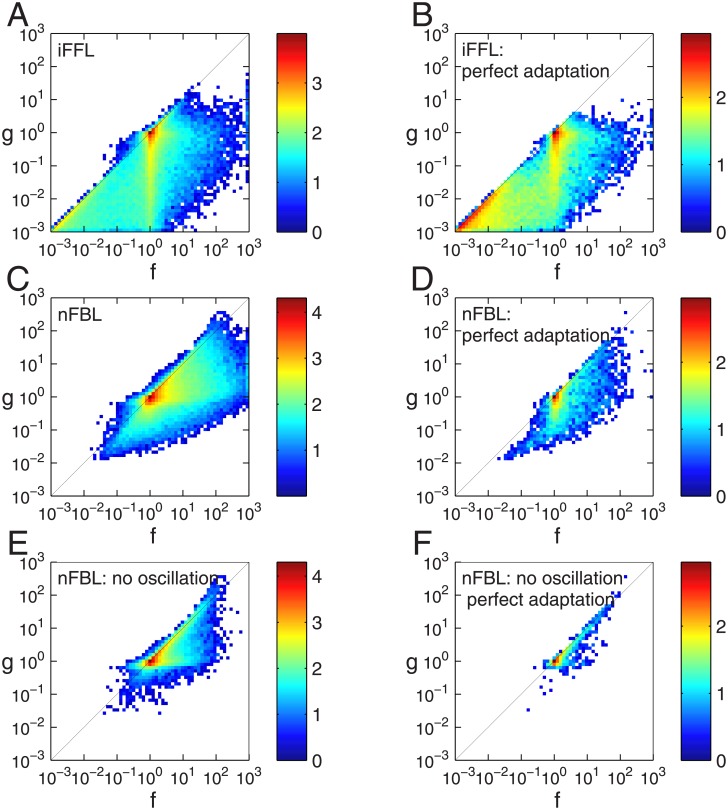
Joint histograms of Fano factor and gain for adaptation networks. The histograms of Fano factor *f* and gain *g* are shown for iFFL (A, B) and nFBL (C-F). Colors indicate the density of parameters in a given region, with red indicating maximum density. (A) iFFL with adaptive response including imperfect adaptation. (B) iFFL with only perfect adaptation. (C) nFBL with adaptive response including imperfect adaptation. (D) nFBL with only perfect adaptation. (E) nFBL with non-oscillatory adaptive response including imperfect adaptation. (D) nFBL with non-oscillatory and only perfect adaptation. The histograms are made for logarithms of both Fano factor *f* and gain *g*. The range of the density is indicated by the colorbar, where the numbers indicate the logarithmic of the density of the parameters. For details, see the section Methods.

The joint histograms of the gain *g* and Fano factor *f* is shown for the parameters with adaptive responses and with perfect adaptation for iFFL and for nFBL in Fig 3. For both iFFL and nFBL, we observe that the gain seem to be not larger than the variance, given by

$$g\leq \frac{\sigma_{a}^{2}}{A^{*}}$$ (15)

indicating that the gain is limited by noise. We note that the average and the variance on the right hand side are considered with respect to the number of molecules rather than concentration. When these values are considered in the unit of concentration, an appropriate pre-factor is necessary to convert the unit of right hand side to a dimensionless number. For iFFL shown in Fig 3(A), the parameters with adaptive responses are distributed in a wide range of both gain and noise strengths from 10^−3^ to 10^1^ satisfying inequality Eq (15). The same tendency is seen even for the parameters with perfect adaptation (Fig 3(B)). For nFBL shown in Fig 3(C), the parameters with adaptive responses are distributed in a wide range with inequality Eq (15). In contrast to iFFL, however, they are rarely distributed in the range with smaller values of both gain and noise. Furthermore, when the adaptation is perfect, as shown in Fig 3(D), the parameters lie relatively closer to the line which satisfies equality in Eq (15). Such a tendency is even more pronounced when we focus on the parameters with only non-oscillatory responses as shown in Fig 3(E) and 3(F).

**Fig 4:**
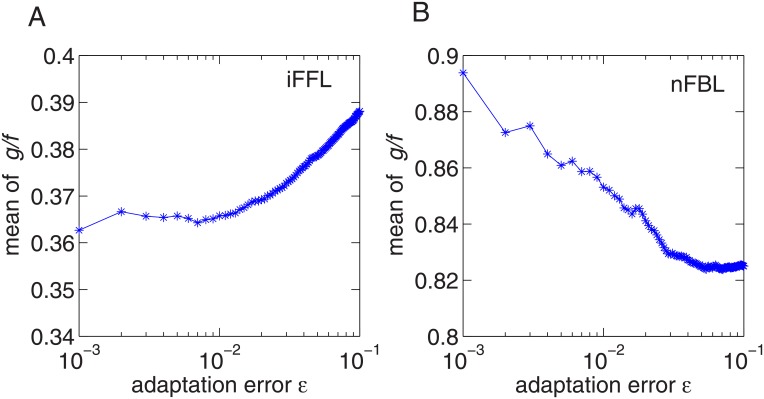
The dependence of the average ratio between gain and Fano factor *g*/*f* on the adaptation error *ϵ*. (A) iFFL (B) nFBL with non-oscillatory response.

To see that with the perfect adaptation condition the points in nFBL (Fig 3F) are distributed closer to the line of equality than in iFFL (Fig 3B), we consider the ratio between the gain and Fano factor, *g*/*f*. In Fig 4, using the samples in Fig 3A and 3E, we plot the average of the ratio *g*/*f* calculated over the samples for which the adaptation error *ϵ* is less than a given threshold. This threshold decreases from 10^−^1 to 10^−^3, implying the selected points show increasing more perfect adaptation. In the plot, the average of ratio *g*/*f* of nFBL is larger than that of iFFL for any value of *ϵ*, indicating that nFBL is closer to the equality line than iFFL. Furthermore, as the perfect adaptation becomes more strict as the adaptation error *ϵ* decreases, the average value of ratio *g*/*f* for nFBL increases approaching to the equality line, whereas that for iFFL decreases. This indicates that in nFBL the perfect adaptation condition takes the gain closer to the limit of Fano factor.

### The adaptive response is limited by noise

Here, we derive the gain-noise inequality given by Eq 15 analytically within a linear range. We first introduce the linear chemical Langevin equation. The perfect adaptation condition is given in terms of the linear coefficients. Then, the gain and noise are derived and compared.

We consider small deviations in *A* and *B* at the steady state values, denoted by *a* and *b*, respectively. By performing the linearization for the chemical Langevin equation Eq (35) shown in Appendix A and applying the linear noise approximation [38] at the steady state, we obtain the linearized Langevin equation given by

$$\begin{array}{c} \dot{a}=-k_{11}a-k_{12}b+\gamma_{a}\Delta S\left(t\right)+\xi_{a}\left(t\right)\\ \dot{b}=-k_{21}a-k_{22}b+\gamma_{b}\Delta S\left(t\right)+\xi_{b}\left(t\right) \end{array}$$ (16)

with the linear coefficient or regression matrix,

$$K=(\begin{array}{cc} k_{11} & k_{12}\\ k_{21} & k_{22} \end{array})$$ (17)

where *γ*_*a*_ and *γ*_*b*_ are the coupling with the input signal *S*, and *ξ*_*a*_ and *ξ*_*b*_ are the Gaussian white noise with ⟨*ξ*_*a*_⟩ = ⟨*ξ*_*b*_⟩ = 0 and ⟨*ξ*_*i*_(*t*)*ξ*_*j*_(*t*′)⟩ = *D*_*ij*_ *δ*(*t* − *t*′), (*i*, *j* = *a*, *b*). Here, ***K***, *γ*_*a*_, *γ*_*b*_ and ***D*** = {*D*_*ij*_} are can be written with the reaction constants, which are explicitly given in Appendix B. Using the eigenvalues of ***K***, *λ*_1_ > 0 and *λ*_2_ > 0, and the corresponding eigenvectors, ^*t*^(1, *p*_1_) and ^*t*^(*p*_2_, 1), ***K*** can be rewritten as ***K*** = ***P*Λ*P*** ^−1^ with

$$P=(\begin{array}{cc} 1 & p_{2}\\ p_{1} & 1 \end{array})\;\text{and}\;\Lambda =\lambda_{1}\;(\begin{array}{cc} 1 & 0\\ 0 & \chi \end{array})$$ (18)

where *χ* = *λ*_2_/*λ*_1_. The regression matrix *K* for iFFL is given with *p*_1_ = 0, while the nFBL is given with *γ*_*b*_ = 0. The perfect adaptation condition that *a* vanishes (*a* = 0) at steady state is given as

$$\gamma_{a}\left(p_{1}-\chi /p_{2}\right)=\gamma_{b}\left(1-\chi \right)$$ (19)

In iFFL, with this perfect adaptation condition, the regression matrix reduces to

$$K=\lambda_{1}\;(\begin{array}{cc} 1 & \frac{\gamma_{a}\chi }{\gamma_{b}}\\ 0 & \chi \end{array}).$$ (20)

Because the eigenvalues *λ*_1_ and *λ*_2_ are always positive real number, the ratio *χ* for iFFL can take any positive value. In the case of nFBL, with the perfect adaptation condition, the regression matrix is given by

$$K=\lambda_{1}(\begin{array}{cc} 1+\chi & -\chi /p_{1}\\ p_{1} & 0 \end{array}).$$ (21)

Here, the eigenvalues can be real or complex. For complex eigenvalues, damping oscillations takes place in the response. For real eigenvalues and non-oscillatory response, the ratio *χ* is constrained to be always not larger than unity, i.e., *χ* ≤ 1.

Next, we derive the gain *g* using the linearized equation Eq (16) and condition Eq (19), which is given by

$$g=\frac{S}{A^{*}}\frac{\gamma_{a}}{\lambda_{1}}\chi^{\frac{\chi }{1-\chi }}$$ (22)

The variance of *a*, $\sigma_{a}^{2}$, is obtained by using the generalized fluctuation-response relation, ***Kσ*** ^2^ + ***σ*** ^2 t^ ***K*** − ***D*** = 0, where ***σ*** ^2^ is the variance-covariance matrix, ***K*** is the regression matrix, and ***D*** is the noise strength. When *D*_*ab*_ = 0, the variance of *a* is calculated as

$$\sigma_{a}^{2}=\frac{D_{aa}\gamma_{b}^{2}+D_{bb}\gamma_{a}^{2}}{2\lambda_{1}{\left(\gamma_{b}-p_{1}\gamma_{a}\right)}^{2}}\frac{\chi }{1+\chi }+\frac{D_{aa}}{2\lambda_{1}\left(1+\chi \right)}$$ (23)

Now, we compare the gain and noise by using Eqs (22) and (23). By comparing the gain and noise, we find that the sufficient condition for the inequality Eq (15) is given by

$$D_{aa}\geq 2S\gamma_{a}\left(1+\chi \right)\chi^{\frac{\chi }{1-\chi }},$$ (24)

As the noise strengths *D*_*aa*_ for both iFFL and nFBL can be written as

$$D_{aa}=2\gamma_{a}S.$$ (25)

the above condition Eq (24) is always satisfied in both network motifs. Therefore, the gain is always less than the intrinsic noise, and it is a direct consequence of the relation between noise strength and input coupling constant for the adapting variable. This relation can be also be extended to the case with *D*_*ab*_ ≠ 0.

### Equality conditions restricts gain around unity in iFFL

We next provide insight into how the equality between gain and noise in Eq (15) are achieved for iFFL. With the condition for iFFL, *p*_1_ = 0, and Eq (25), the difference between gain and noise is given by

$$\frac{\sigma_{a}^{2}}{A^{*}}-g=\frac{D_{bb}\gamma_{a}^{2}}{2\lambda_{1}A^{*}\gamma_{b}^{2}}\frac{\chi }{1+\chi }+\frac{\gamma_{a}S}{\lambda_{1}A^{*}}-\frac{\gamma_{a}S}{\lambda_{1}A^{*}}\chi^{\frac{\chi }{1-\chi }}$$ (26)

The second term on the right hand side indicates the contribution of noise generated in reactions of A. Because it does not pass through reactions in B, the second term is independent of the ratio *χ* that includes the property of reaction in B. We also notice that since the second term is independent of B, it does not depend on whether the adaptation is perfect or not. In Eq (26), as *χ* decreases to zero, the second and third terms are canceled with each other, while the first term vanishes. As a result, the deviation between gain and noise vanishes, and the equality in Eq (15) holds. In contrast, as *χ* increases, the first term increases, while the deviation between the second and third terms increases.

**Fig 5:**
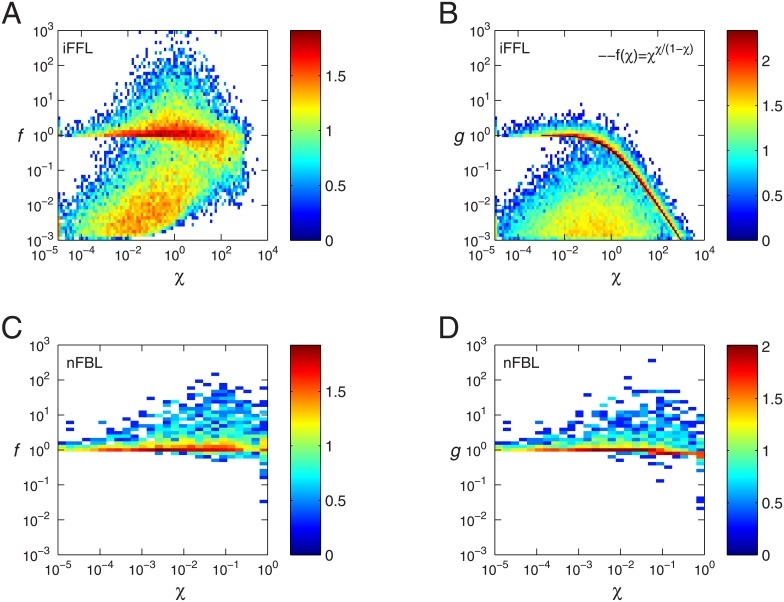
The dependence of gain and noise on the ratio of eigenvalues. The joint histograms of the Fano factor *f* and ratio *χ* of eigenvalues (A, C), and the gain *g* and *χ* (B, D) for iFFL (A, B) and nFBL (C, D), for the parameters with perfect adaptation. For nFBL, the non-oscillatory case is considered. The colors indicate the log of histogram of the density of points, as explained in Fig 3. The dashed line in (B) shows $f(\chi )=\chi^{\frac{\chi }{1-\chi }}$.

We verified this dependence on *χ* numerically, as shown in Fig 5(A) and 5(B). As *χ* decreases, both gain and noise approach to unity, reaching the equality. In contrast, as *χ* increases, the gain systematically decreases as $\chi^{\frac{\chi }{1-\chi }}$ according to Eq (22), while the Fano factor *f* is maintained around unity or larger than unity for many parameter sets (Fig 5(A)) independent of *χ*, because the noise contribution from the reactions of A is independent of *χ*. As a result, the deviation between noise and gain increases with a larger value in Eq (26).

**Fig 6:**
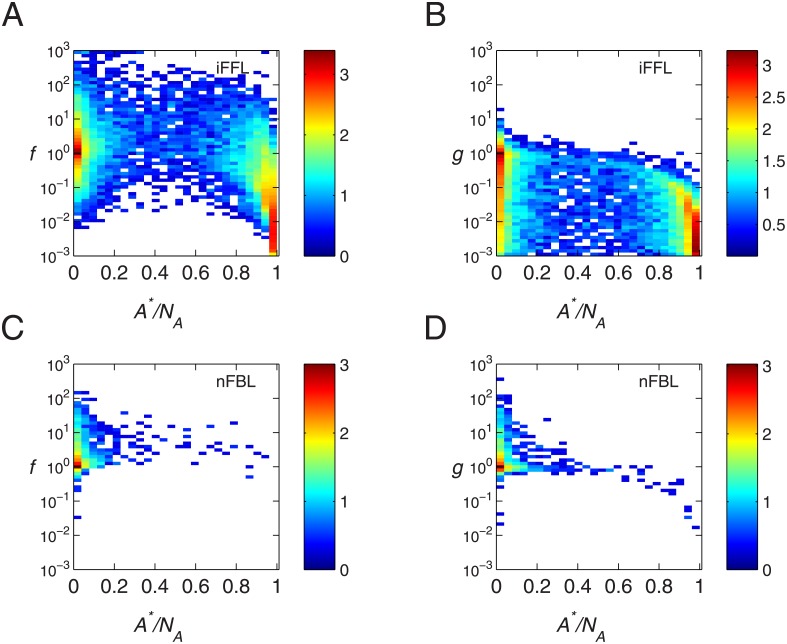
The dependence of Fano factor and gain on the number in steady state. The scatter plot for steady state number A*/*N*_*A*_ with Fano factor *f* and gain *g* have been plotted for the parameters showing perfect adaptation. Color indicates the density of parameters as explained in the Fig 3. (A) A*/*N*_*A*_ against *f* for iFFL. (B) A*/*N*_*A*_ against *g* for iFFL (C) A*/*N*_*A*_ against *f* for nFBL. (B) A*/*N*_*A*_ against *g* for nFBL.

For iFFL, the number A* can take any values up to *N*_*A*_ without any constraint by the perfect adaptation condition. When A* is large and close to *N*_*A*_, the noise (the first two terms in Eq (26)) and the gain (the third term) decrease as *A* approaches to *N*_*A*_, because the response coefficient $\gamma_{a}=\Gamma_{\text{a}}^{\text{A}}(N_{A}-A^{*})$ decreases as A* approaches to *N*_*A*_. This dependence of gain and noise on A* is also found numerically as shown in the Fig 6(A) and 6(B). Thus, as A* approaches to *N*_*A*_, the gain approaches to the noise, though the gain is small.

Another way to achieve the equality in Eq (15) is to decrease *χ*. When A*/*N*_*A*_ ≪ 1, the ratio *χ* can be approximated as

$$\chi \approx \frac{-p_{2}}{\gamma_{a}\gamma_{b}}\approx \frac{\frac{K_{\text{SB}}}{K_{\text{SB}}+N_{B}}\Gamma_{a}^{B}+\frac{\Gamma_{d}^{B}}{S}}{\frac{K_{\text{SA}}}{K_{\text{SA}}+N_{A}}\frac{\Gamma_{a}^{A}}{\Gamma_{d}^{A}}\frac{\Gamma_{d}^{B}}{\Gamma_{a}^{B}N_{B}}+1}\frac{\Gamma_{d}^{B}}{\Gamma_{a}^{B}N_{B}}\frac{1}{\Gamma_{d}^{A}}$$ (27)

For this condition, the coefficient of gain in Eq (22) and the second term in Eq (26) are approximately given by

$$\begin{array}{ccc} \frac{\gamma_{a}S}{\lambda_{1}A^{*}} & \approx & 1/(\frac{K_{\text{SA}}}{K_{\text{SA}}+N_{A}}\frac{\Gamma_{a}^{A}}{\Gamma_{d}^{A}}\frac{\Gamma_{d}^{B}}{\Gamma_{a}^{B}N_{B}}+1) \end{array}$$ (28)

which can increase up to unity as the first term in the denominator decreases. By increasing $\Gamma_{d}^{\text{A}}$ in Eqs (27) and (28), *χ* can decrease systematically approaching the equality condition in Eq (15), while both gain *g* and the Fano factor *f* approach to unity. Therefore, as shown in Fig 3(C), with perfect adaptation property, the equality between gain and noise is achieved when both values are about unity.

### Perfect adaptation decreases the noise in nFBL

For nFBL with non-oscillatory responses, we consider the conditions under which the gain approaches to the equality in Eq (15). With the condition *γ*_*b*_ = 0 for nFBL, and Eq (25), the difference between gain and noise is given by

$$\frac{\sigma_{a}^{2}}{A^{*}}-g=\frac{D_{bb}}{2\lambda_{1}A^{*}p_{1}^{2}}\frac{\chi }{1+\chi }+\frac{\gamma_{a}S}{\lambda_{1}A^{*}}\frac{1}{1+\chi }-\frac{\gamma_{a}S}{\lambda_{1}A^{*}}\chi^{\frac{\chi }{\chi -1}}$$ (29)

In contrast to iFFL, the second term on the right hand side is a function of *χ*, because the noise contribution from reactions of A is affected by reactions of B. Because *χ* ≤ 1 is always satisfied as mentioned above, the second and third terms are easier to be close to each other than iFFL. In fact, as shown in Fig 5(C) and 5(D), the gain and noise exhibit similar tendency for any value of *χ*. Thus, the equality in Eq (15) is achieved easier than iFFL. When *A* is sufficiently smaller than the total number *N*_*A*_, the coefficient in the second and third terms is approximately given by

$$\begin{array}{ccc} \frac{\gamma_{a}S}{\lambda_{1}A^{*}} & \approx & \frac{1+A^{*}/K_{\text{BA}}}{\frac{K_{\text{SA}}}{K_{\text{SA}}+N_{A}}\frac{A^{*}}{N_{A}}\left(1+A^{*}/K_{\text{BA}}\right)+1} \end{array}$$ (30)

which can be larger than unity. This indicates that even for sufficiently large *χ* approaching to zero on the right hand side in Eq (29), a higher gain is possible. For non oscillatory parameters and for adaptive response, the dependence of gain and noise on the steady state is shown in Fig 6(C) and 6(D). In contrast to iFFL, steady state values close to *N*_*A*_ don’t occur in nFBL.

In Fig 3(E) without perfect adaptation conditions, the points are present far below the equality line. Among all points shown in Fig 3(E), only points that exhibit perfect adaptation are shown in Fig 3(F). As mentioned above, those points with perfect adaptation are distributed closely to the equality line. To study this behavior, we introduce the deviation from the perfect adaptation, *ω*, given by

$$\omega =\frac{p_{1}p_{2}-\chi }{1-\chi }.$$ (31)

From Eq (19), the perfect adaptation condition is given by *ω* = 0. Then, the gain and the noise in A are respectively written as

$$g=\frac{\gamma_{a}S}{\lambda_{1}A^{*}}\frac{{\left(\chi +\omega \left(1-\chi \right)\right)}^{\frac{1}{1-\chi }}-\omega }{\chi \left(1-\omega \right)}$$ (32)

and

$$\frac{\sigma_{a}^{2}}{A^{*}}=\frac{D_{bb}{\left(\chi +\omega \left(1-\chi \right)\right)}^{2}}{\lambda_{1}A^{*}p_{1}^{2}\chi \left(1+\chi \right){\left(1-\omega \right)}^{2}}+\frac{\gamma_{a}S}{\lambda_{1}A^{*}}\frac{\chi {\left(1-\omega \right)}^{2}+\omega^{2}}{\chi \left(1+\chi \right){\left(1-\omega \right)}^{2}}$$ (33)

**Fig 7:**
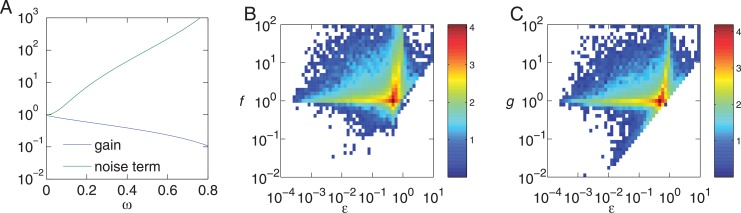
Dependence of Fano factor and gain on the adaptation error. Gain and noise as a function of adaptation error for nFBL has been plotted. (A) Gain and Fano factor as a function of deviation from perfect adaptation *ω* calculated from Eqs (32) and (33) for *χ* = .01 and $\frac{\gamma_{a}S}{\lambda_{1}A^{*}}=1$. (B) Fano factor as a function of *ϵ* for nFBL obtained from simulations of different parameters, plotted as a scatter plot with the colors indicating the logarithm of density of parameters, as explained in Fig 3. (C) Gain as a function of *ϵ* for different parameters of nFBL. Note that while both *ϵ* and *ω* denote the deviation from adaptation, their definitions are slightly different. From simulation, the parameters showing non oscillatory response only have been included.

In Fig 7(A), the gain in Eq (32) (blue line) and the second term in the noise in Eq (33) (green line) are plotted as functions of *ω*. When *ω* = 0 and the perfect adaptation condition is satisfied, these two values are close to each other. As *ω* increases, the gain decreases while the noise increases. Therefore, in nFBL the perfect adaptation condition can contribute to increase the gain and decrease the noise at the same time. In Fig 7(B) and 7(C), we show the effect of adaptation error on the noise and gain for nFBL. As the adaptation error decreases, the gain and noise both approach same value, thus leading to high signal to noise ratio in the limit of perfect adaptation.

## Discussion

In this paper, we have studied the relation between response and noise in the network motifs that show perfect adaptation. By numerical simulation, we found that the gain measured at the peak response is not larger than the intrinsic noise, measured as the Fano factor. For the case with perfect adaptation, we showed analytically that the inequality in Eq (15) holds in the linear regime. In iFFL, modulating the gain towards its limiting value of Fano factor constraints both of them around a range of unity. Thus, for iFFL, parameters showing perfect adaptation, higher gain and unity gain to noise ratio is difficult to achieve. In contrast, for nFBL, while imperfect adaptation itself shows higher gain to noise ratio, perfect adaptation narrows down the range of gain around its limit of Fano factor. We also note that in nFBL the parameters that show perfect adaptation property also exhibit higher gains. In contrast, in iFFL the parameters with perfect adaptation are distributed in a range from low values to around unity, though the frequency of perfect adaptation among randomly chosen parameters are higher than that in nFBL.

We can have some insight about the differences between iFFL and nFBL for perfect and imperfect adaptation by looking at the analytical terms of the response function and the correlation function. Between the correlation function ***C***(*t*) and the response function ***h***(*t*) = exp(−***K*** *t*), the equation $\frac{dC(t)}{dt}=-h(t)\frac{D+\alpha }{2}$ holds, where ***α*** characterizes the degree of non-equilibrium defined as ***Kσ*** ^2^ − ***σ*** ^2 t^ ***K*** − ***α*** = 0 [39]. By integrating this equation with respect to time *t*, and introducing a step response function ***R***, which is given by $\text{R}(t)=\int_{0}^{t}\text{h}(s)ds$, we have

$$\frac{1}{2}R\left(t^{*}\right)D+\frac{1}{2}R\left(t^{*}\right)\alpha =\sigma^{2}-C\left(t^{*}\right)$$ (34)

at time t* = log *χ*/*λ*_1_(*χ* − 1), which is the time when the response in *A* reaches the maximum value. The response in *A* to a step increase in *S* is given by the first row of ***R***(t*) ⋅ **γ**, i.e., $g=\frac{S}{A^{*}}(1,0)\cdot \text{R}(t^{*})\cdot \text{γ}$. For iFFL, considering $\text{γ}=\frac{1}{2S}\text{D}\cdot {\;}^{t}\;(1,1)$, the gain *g* can be calculated from the first term on the left hand side in Eq (34) by applying ^*t* (1,1)^ to Eq (34) from the right. When the ratio of eigenvalues, *χ*, is sufficiently small, the (1,2) component in ***R*** vanishes as evident from the regression matrix ***K*** in Eq (20). Thus, only (1,1) component in ***R***(t*) contributes to the gain *g*. We also note that the diagonal components in **α** is always zero by the definition, indicating that the second term does not contribute to the gain *g*. On the right hand side in Eq (34), **C**(t*) vanishes as *χ* approaches to zero. Consequently, we see that the equality holds in Eq (15), when *χ* is sufficiently small for iFFL. This does not depend on whether the adaptation is perfect. In a similar way, for nFBL, considering $\text{γ}=\frac{1}{2S}\text{D}\cdot {\;}^{t}\;(1,0)$, the (1,1) components in Eq (34) are enough to calculate the gain *g*. As seen in Fig 5(C) and 5(D), even when *χ* is not very small, the gain and Fano factor are close with each other. This indicates that (1,1) component in the first terms in Eq (34) are close to each other while the (1,1) components in second terms are almost canceled at *t* = t*. The condition that this occurs in Eq (34) and the gain approaches to the limit of Fano factor remains to be a future problem.

We have studied the noise generated by the adaption reaction (intrinsic noise). For the noise in an input stimulus (extrinsic noise), filtering properties of adaptive response has been studied [33, 34]. Fast fluctuations in the input are averaged out by the low pass filtering effect, while slow fluctuations are attenuated by adaptation. Thus, adaptive reactions behaves as a bandpass filter. Because of this filtering property, the magnitude of the extrinsic noise is dominated by the intrinsic noise.

In the bacterial chemotaxis, the adaptive responses is well studied. The mechanism of adaptation is considered to be negative feedback loop, in which the receptor activity shows response to ligand-binding, which is then attenuated through a modulation of methylation level by methylesterase CheB, the activity of which depends on the receptor activity. Examples of iFFL has been reported for eukaryotic cells. In a cultured mammalian cell, epidermal growth factor (EGF) induces transient activation of extracellular-signal-regulated kinase (ERK). To explain this adaptive response, an iFFL mechanism upstream of ERK has been proposed, in which phosphorylated EGF receptors activate both Ras GEF and GAP with different time constants [40]. Similarly, for the adaptive response of chemotaxis signaling pathway in *Dictyostelium* cell, iFFL of Ras and its GAP and GEF has been proposed [20].

Previously, it has been reported that iFFL is more robust than nFBL with respect to the perfect adaptation [18]. Our analysis also confirms that the frequency of perfect adaptation is higher in iFFL than nFBL. However, our result indicates that the frequency to have higher gain is larger for nFBL. Moreover, in nFBL the gain is close to the upper limit of the Fano factor, while in iFFL the parameters are broadly distributed away from the upper limit. Also, our analysis shows that the perfect adaptation condition itself can contribute to increase the gain up to the limit of Fano factor for nFBL, which could be of great advantage. In particular, in small cells with smaller number of molecules, such as in bacteria, nFBL should work more robustly against molecular noise. We hope our results would help to understand the mechanism and occurrence of adaptive networks in biological systems.

## Methods

### Sampled parameter space

To quantify the gain and noise for adaptive networks, the Michaelis-Menten parameters for the iFFL and nFBL were sampled randomly, where each of the network needs 8 parameters. The Michaelis-Menten parameters *V*_*m*_ and *K*_*m*_ were sampled from a uniform distribution on a logarithmic scale, from the range (.001 − 1000), where *V*_*m*_ is the dimensionless maximal velocity, and *K*_*m*_ is the Michaelis constant, in the units of moles.

For calculating the deterministic gain using the differential equations, the simulations were performed with the concentration of each species. For computing the Fano factor, and for observing the stochastic response, the simulations were performed with the actual number of molecules with a uniform system size or volume *V* = 10,000 for each parameter. The Michaelis constant in terms of number of molecules, *K*_M_ is computed by *K*_M_ = *VK*_*m*_. The maximum velocity in terms of number of molecules is the same as in the concentration space, that is *V*_M_ = *V*_*m*_.

### Quantifying the response

For a given parameter, first the steady state of the system was obtained. For iFFL, the steady state number can be obtained analytically in a closed form, while for nFBL, the steady state number was obtained using numerically solving the nonlinear differential equation using the Runge-Kutta method. If the steady state number was too small or large, that is (*A*/*V* < 0.001, *B*/*V* < 0.001), or ((*N*_*A*_ − *A*)/*V* < 0.001, (*N*_*B*_ − *B*)/*V* < 0.001), where *V* = 10,000 then we call it an ill-behaved parameter and discard it.

For parameters which were not ill-behaved, a small perturbation Δ*S* was applied to it in the steady state, and the deterministic part of the response was obtained using the differential equation. For solving the differential equation, we used the *fourth order Runge-kutta* method with a default step size of *h* = .001 and the global error tolerance, that is the difference between successive values (*y*_*n*+1_ − *y*_*n*_), to be less than *h*/50, where *y*_*i*_ is the numerical solution of the differential equation at the *i* ^*th*^ step. If the solver failed to reach the steady state with this step size, we successively used a smaller step size of *h* = 10^−4^ or 10^−5^. A response was considered to be perfectly adaptive if the adaptation error *ϵ* < 0.01. The response was calculated in the concentration space and then converted into numbers using the system volume information.

From the obtained time series of the response, the quantities A*, the initial steady state, *A*_max_, the maximum number, and A**, the final number of the node A was obtained. The maximum change in the output is Δ*A* = (*A*_max_ − A*). Using these quantities, we compute the following:

1. Adaptive tendency $\alpha =\frac{A_{\text{max}}-A^{**}}{A_{\text{max}}-A^{*}}$, which quantifies the tendency of the network to get back to its pre stimulus state from its maximum state.
2. Adaptation error $\epsilon =\frac{(A^{**}-A^{*})/A^{*}}{\Delta S/S}$, which quantifies the deviation from perfect adaptation.
3. Gain $g=\frac{|\Delta A|/A^{*}}{\Delta S/S}$, which gives a scale free estimate of the response for a perturbation of Δ*S* in the input signal.

For nFBL, the oscillatory and non oscillatory parameters were differentiated by observing the response time series, where the total number of times the slope of the response changed its sign was calculated. If the total sign change happened at least three times, then we considered the response as oscillatory response.

### Quantifying the Noise

To quantify the Fano factor, the system was simulated by using the stochastic simulation algorithm described by Gillespie [37]. All the simulations were performed with a system volume of *V* = 10,000, and reaction was ran for a minimum of 100 time units and 10^5^ reaction events. The steady state of the system itself was chosen as the initial condition. The resultant time series was used to calculate the variance *σ* ^2^ of the output. From this output, the Fano factor can be estimated by $f=\frac{\sigma^{2}}{A^{*}}$ as defined in Eq (12) where A* is the steady state value.

#### Parameters used for Fig 2

The exact parameters used for the time series plot in the Fig 2 are as follows:

- Fig 2A: *V*_sa_ = 0.19, *K*_sa_ = 6, *V*_ba_ = 57, *K*_ba_ = 0.007, *V*_sb_ = 0.01, *K*_sb_ = 10, *V*_b_ = 119, *K*_b_ = 55.9
- Fig 2B: *V*_sa_ = 0.02, *K*_sa_ = 0.04, *V*_ba_ = 8.9, *K*_ba_ = 1.5, *V*_sb_ = 0.29, *K*_sb_ = 3.7, *V*_b_ = 651, *K*_b_ = 53.
- Fig 2C: *V*_sa_ = 1.03, *K*_sa_ = 0.008, *V*_ba_ = 11.3, *K*_ba_ = 3.22, *V*_ab_ = 0.29, *K*_ab_ = 0.002, *V*_b_ = 0.17, *K*_b_ = 0.004
- Fig 2D: *V*_sa_ = 0.0035, *K*_sa_ = .63, *V*_ba_ = 0.003, *K*_ba_ = 0.001, *V*_ab_ = 2.79, *K*_ab_ = 9.5, *V*_b_ = 0.2, *K*_b_ = 0.5

The total number of molecules was *N*_A_ = *N*_B_ = 200,000, *S* = 10,000, and a large perturbation of Δ*S* = 10000 was provided at the steady state, where the volume is *V* = 10,000.

#### Parameters for Fig 3 and following figures

For obtaining the joint histogram of Fano factor and gain in Fig 3, a total of 10^6^ random parameters were sampled for each of iFFL and nFBL, and both Fano factor and gain was calculated for them. In each of the above case, the total number of molecules was *N*_A_ = *N*_B_ = 200,000, *S* = 10,000, and a 1% perturbation of Δ*S* = 100 was provided at the steady state, where the volume is *V* = 10,000. The same parameters were also used for the Figs (4–7) as well.

### Making the log histogram plot

To represent the distribution of Fano factor and gain or for other pair of variables, we used log of the histogram plot, as there was large variation in the parameter density in low density and high density regions. To obtain the log histogram plot, first the relevant pair of variables were converted on a log base(base 10). Subsequently, the histogram was obtained by counting the number of parameters falling in each square bin of chosen size. This count was again taken on a log 10 base, and the resultant matrix was plotted on a pseudo color plot using the function *pcolor* of matlab, with maximum density being assigned the red color, and the minimum density as blue color. The binwidths were typically chosen to be of size.1 for both *x* and *y* coordinates.

## Appendix A

Here, we show the chemical Langevin equations for iFFL and nFBL, which are given by

$$\begin{array}{ll} \dot{A} & =\Gamma_{\text{a}}^{\text{A}}S(N_{\text{A}}-A)-\Gamma_{\text{d}}^{\text{A}}BA+\xi_{a}(t)\\ \dot{B} & =\Gamma_{\text{a}}^{\text{B}}S(N_{\text{B}}-B)-\Gamma_{\text{d}}^{\text{B}}B+\xi_{b}(t) \end{array}$$ (35)

and

$$\begin{array}{ll} \dot{A} & =\Gamma_{\text{a}}^{\text{A}}S(N_{\text{A}}-A)-\Gamma_{\text{d}}^{\text{A}}BA+\xi_{a}(t)\\ \dot{B} & =\Gamma_{\text{a}}^{\text{B}}A(N_{\text{B}}-B)-\Gamma_{\text{d}}^{\text{B}}B+\xi_{b}(t) \end{array}$$ (36)

respectively, where $\Gamma_{a}^{\text{A}}$, $\Gamma_{d}^{\text{A}}$, $\Gamma_{a}^{\text{B}}$ and $\Gamma_{d}^{\text{B}}$ are the reaction rates as defined in Eq (3), and *ξ*_*a*_ and *ξ*_*b*_ are the Gaussian white noise with ⟨*ξ*_*a*_⟩ = ⟨*ξ*_*b*_⟩ = 0 and ⟨*ξ*_*i*_(*t*)*ξ*_*j*_(*t*′)⟩ = *D*_*ij*_ *δ*(*t* − *t*′), (*i*, *j* = *a*, *b*). The noise strength ***D*** = {*D*_*ij*_} can be derived from the property of Poisson process in individual reactions [38, 41]. Thus, *D*_*ij*_ are given by $D_{aa}=2\Gamma_{d}^{\text{A}}A\cdot B$, $D_{bb}=2\Gamma_{d}^{\text{B}}B$, and *D*_*ab*_ = *D*_*ba*_ = 0.

## Appendix B

Here, we show the explicit forms of the linear coefficients ***K***, **γ** and ***D*** in the linearized Langevin equations Eq (16). For iFFL, the liner coefficients ***K*** in Eq 35 are given by

$$K=(\begin{array}{cc} \frac{V_{\text{SA}}K_{\text{SA}}S}{{\left(K_{\text{SA}}+N_{A}-A^{*}\right)}^{2}}+\frac{V_{\text{BA}}K_{\text{BA}}B^{*}}{{\left(K_{\text{BA}}+A^{*}\right)}^{2}} & \frac{V_{\text{BA}}A^{*}}{K_{\text{BA}}+A^{*}}\\ 0 & \frac{V_{\text{SB}}K_{\text{SB}}S}{{\left(K_{\text{SB}}+N_{B}-B^{*}\right)}^{2}}+\frac{V_{B}K_{B}}{{\left(K_{B}+B^{*}\right)}^{2}} \end{array})$$ (37)

The coupling coefficients **γ** with the input *S* also are obtained in the above step, which are given by

$$\left(\begin{array}{c} \gamma_{a}\\ \gamma_{b} \end{array})=(\begin{array}{c} \frac{V_{\text{SA}}\left(N_{A}-A^{*}\right)}{K_{\text{SA}}+N_{A}-A^{*}}\\ \frac{V_{\text{SB}}\left(N_{B}-B^{*}\right)}{K_{\text{SB}}+\left(N_{B}-B^{*}\right)} \end{array}\right)$$ (38)

For the nFBL, using the Eq (35), ***K*** is given as

$$K=(\begin{array}{cc} \frac{V_{\text{SA}}K_{\text{SA}}S}{{\left(K_{\text{SA}}+N_{A}-A^{*}\right)}^{2}}+\frac{V_{\text{BA}}K_{\text{BA}}B^{*}}{{\left(K_{\text{BA}}+A^{*}\right)}^{2}} & \frac{V_{\text{BA}}A^{*}}{K_{\text{BA}}+A^{*}}\\ \frac{V_{\text{AB}}\left(N_{B}-B^{*}\right)}{K_{\text{AB}}+N_{B}-B^{*}} & \frac{V_{\text{AB}}K_{\text{AB}}}{{\left(K_{\text{AB}}+N_{B}-B^{*}\right)}^{2}}+\frac{V_{B}K_{B}}{{\left(K_{B}+B^{*}\right)}^{2}} \end{array})$$ (39)

The coupling coefficient **γ** with the input *S* is obtained and given by:

$$\left(\begin{array}{c} \gamma_{a}\\ \gamma_{b} \end{array})=(\begin{array}{c} \frac{V_{\text{SA}}\left(N_{A}-A^{*}\right)}{K_{\text{SA}}+N_{A}-A^{*}}\\ 0 \end{array}\right)$$ (40)

The noise strength ***D*** can be obtained by the linear noise approximation [38].

$$D=(\begin{array}{cc} 2\Gamma_{d}^{A}A^{*}B^{*} & 0\\ 0 & 2\Gamma_{d}^{B}B^{*} \end{array})=(\begin{array}{cc} 2\frac{V_{\text{BA}}B^{*}A^{*}}{K_{\text{BA}}+A^{*}} & 0\\ 0 & 2\frac{V_{B}B^{*}}{K_{B}+B^{*}} \end{array})$$ (41)
